# A Cost-Effectiveness Analysis to Evaluate a System Change in Mental Healthcare in the Netherlands for Patients with Depression or Anxiety

**DOI:** 10.1007/s10488-017-0842-x

**Published:** 2017-12-15

**Authors:** Kasper van Mens, Joran Lokkerbol, Richard Janssen, Mirjam L. van Orden, Margot Kloos, Bea Tiemens

**Affiliations:** 1 Altrecht Mental Health, Utrecht, The Netherlands; 2 Centre of Economic Evaluation Trimbos Institute (The Netherlands Institute of Mental Health and Addiction), Utrecht, The Netherlands; 3 Rob Giel Research Centre, University Medical Centre Groningen, Groningen, The Netherlands; 4 Tilburg University, Tilburg, The Netherlands; 5 Erasmus University Rotterdam, Rotterdam, The Netherlands; 6 Parnassia Groep, Parnassia Academie, The Hague, The Netherlands; 7 Pro Persona Research, Pro Persona, Wolfheze, The Netherlands; 8 Indigo Service Organization, Utrecht, The Netherlands; 9 Radboud University, Nijmegen, The Netherlands

**Keywords:** Cost-effectiveness, Depression, Anxiety

## Abstract

Over the last decade, the Dutch mental healthcare system has been subject to profound policy reforms, in order to achieve affordable, accessible, and high quality care. One of the adjustments was to substitute part of the specialized care for general mental healthcare. Using a quasi-experimental design, we compared the cost-effectiveness of patients in the new setting with comparable patients from specialized mental healthcare in the old setting. Results showed that for this group of patients the average cost of treatment was significantly reduced by, on average, €2132 (p < 0.001), with similar health outcomes as in the old system.

## Background

Over the last decade, significant changes have been implemented in the organization and financing of the mental healthcare system in the Netherlands (Nas and Van Geldrdop 2013; Westra et al. 2016). The reforms started with the introduction of regulated competition, in which service providers have to negotiate with health insurers on both costs and quality of care. The overall goal of the reforms was to realise a national healthcare system that is accessible for every citizen, is affordable, and improves the quality of care.

One of the reforms was de introduction of the so-called basic mental healthcare segment, targeting those patients with a mental disorder who could be helped with relatively short term generic interventions, rather than with the more specialized treatments in the specialized mental healthcare segment. The defining feature of the new basic mental healthcare segment was that it introduced short treatment products, limited to roughly five, eight, or twelve sessions. This, in contrast to specialized mental healthcare, where treatments can be as short as five sessions, but are not limited in duration. The General Practitioner (GP) serves as a gatekeeper to both segments, referring patients to either basic mental healthcare or specialized mental healthcare. This means that it is not possible for patients to transfer freely between the segments. Other than that, the organization and financing structure of both segments are comparable, with for example treatments requiring co-payments from patients in both segments.

Basic mental healthcare was intended to serve a subset of patients who would previously have been treated in specialized mental healthcare. This subset includes patients with mild to severe mental health problems and with low to moderate complexity, and with low risk of suicide or dangerous behaviour. Due to the limited treatment duration in basic mental healthcare, it is likely (but not necessarily the case) that the cost per patient is lower in basic mental healthcare. However, it is not known whether restricting the treatment duration comes with a loss of effectiveness.

This study is a cost-effectiveness analysis of basic healthcare in comparison with specialized mental healthcare, in which equivalent patients from before and after the introduction of basic mental healthcare are compared.

## Methods

### Setting

In this study we used routine administrative data (from a nationwide franchised company) from a two year period after the introduction of basic mental healthcare, as well as data (from two large specialized mental healthcare agencies) from a 3 year period before the introduction of basic mental healthcare. The two large specialized mental healthcare agencies have multiple sites in both urban and rural areas. The patient population consists of people of all ages and psychiatric disorders, and the agencies offer a broad range of specialized inpatient and outpatient treatment. The nationwide franchised company consists of 200 locations scattered over a large part of the Netherlands, also in both urban and rural areas. The company offers only outpatient basic mental healthcare products for patients with all kinds of disorders. Depression and anxiety disorders are the most common disorders in basic mental healthcare.

### Design

Because this study was performed in a naturalistic setting, the most rigorous design that could be used was a historical matched cohort study. The study used real data, which was generated and stored during the execution of treatment, and was used for reimbursement. Pre- and post-treatment test scores were used to analyse treatment effects, and reimbursement data was used to determine costs. Costs and effects over the restricted treatment duration in the basic mental healthcare setting were compared with costs and effects in the treatment period in the specialized mental healthcare setting.

### Patient Cohorts

The first sample included patients from specialized mental healthcare whose treatment started in the years 2011, 2012, or 2013. The second sample included patients from basic mental healthcare whose treatment started in 2014 or 2015. To increase comparability between the samples, only patients were included who:

- received outpatient care for a main diagnosis of depression or anxiety disorder,
- had not received earlier treatment for their mental disorder in that mental healthcare setting (first treatment),
- had valid ROM test scores at the start and at the end of treatment,
- were not defined as chronically ill.

In order to deal with overt selection bias, a propensity score matching algorithm was used to select patients from both samples (Austin 2011a). Adjustment for remaining confounding differences was done by using a regression analysis to assess the differences in cost and effect. Findings are reported in line with the Consolidated Health Economic Evaluation Reporting Standards (CHEERS) (Husereau et al. 2013).

### Data Collection

Data was collected for the period from 2011 until 2013 from the two large mental healthcare agencies, and for the period from 2014 until 2016 from the franchised company. The registration data included all activities performed during the treatment from diagnostic intake to discharge. This data exactly described the amount of time that each type of healthcare professional was involved. Routine Outcome Monitoring (ROM) data was collected to determine the primary clinical outcome of a treatment. Only patients with no missing data were included in the analysis.

### Health Outcome

The primary clinical outcome was defined as the standardized improvement in level of symptom severity between the start and the end of a treatment. Symptom severity was measured with a ROM questionnaire at several evaluation moments in the treatment. In the participating mental healthcare agencies and the franchised company, different instruments were used to measure symptom severity. Therefore, the difference in test score was converted to a Cohen’s d effect size (Cohen 1988). The ROM instruments used in our analyses were the symptom-distress scale of the Outcome Questionnaire (OQ-45.2) and the short symptom list (KKL, Korte Klachten Lijst) (Lambert et al. 2004; Lange and Appelo 2007).

### Instruments

The KKL is a self-report questionnaire that measures the degree of suffering from common mental health problems such as anxiety, depression, eating disorders, sleeping problems, and addiction (van de Ven et al. 2000). The 13 items of the questionnaire are scored on a 5-point Likert scale, ranging from 0 (not suffering at all) to 4 (a vast amount of suffering). Psychometric properties of the KKL were acceptable to good (Lange and Appelo 2007; van de Ven et al. 2000).

The OQ-45.2 is a standardized self-report outcome measure designed for repeated measurement of client progress in therapy, and assesses problems relating to anxiety, depression, and substance abuse (Lambert et al. 2004). The 25 items of the Symptom Distress (SD) subscale of the OQ-45.2 are scored on a 5-point Likert scale, ranging from 0 (never) to 4 (almost always). Psychometric properties of the Dutch OQ-45.2 were adequate (De Jong et al. 2007).

### Costs

The costs of each treatment were calculated from a healthcare perspective and included staff costs, overhead, and employer’s contribution, in accordance with the Dutch Guideline on economic evaluations in healthcare (Hakkaart-van Roijen et al. 2015). The costs of treatment were estimated using volume × cost per unit, where volume is equal to the hours of involvement of each type of healthcare professional, and cost per unit is the gross annual salary of each type of healthcare professional divided by 1300 billable hours. In accordance with the guideline, we took the median gross annual salary scale of each type of medical staff from the collective agreements of Dutch mental health services of 2015. The same salary scale was used for both samples and we did not adjust for inflation. According to the guidelines, an increment of 35% on the costs for employer’s contribution was used, and another increment of 38% for the overhead costs. Table 1 presents the unit cost for each type of healthcare professional in the dataset.

**Table 1:** Unit cost overview

	Unit cost (€)
Clinical psychologist	98.19
Social worker	55.09
Basic doctor	81.61
Psychotherapist	81.61
Psychiatrist	129.29
Nurse	43.62
Psychiatric nurse	62.05
Mental health psychologist	81.61
Basic psychologist	70.63
Unknown	82.94
Other	58.33

The unit cost is the median salary of each type of healthcare professional according to the collective agreement of Dutch mental healthcare 2015, with an increment of 35% employer’s contribution and 38% for overhead, divided by 1300 billable hours

### Cost-Effectiveness Analysis

The cost-effectiveness results are presented as an incremental cost-effectiveness ratio (ICER). The ICER describes the incremental costs for one additional health effect gained in basic mental healthcare treatment as compared to specialized mental healthcare treatment (Husereau et al. 2013). One additional health effect gained is defined as standardized improvement in level of symptom severity (expressed in effect size d) measured with the ROM-instruments as described under Health Outcome.

### Statistical Analyses

All of our statistical analyses were performed in R, an open source statistical programming environment (R Core Team 2016). A propensity score matching algorithm from the R package ‘matching’ was used to balance the distribution of five known covariates: gender, age, baseline symptom severity, diagnosis group, and country of origin (Austin 2011a; Sekhon 2011). Because symptom severity was measured with different instruments, baseline symptom severity score was converted to a Z-score. Country of origin was clustered into three categories: the Netherlands, abroad (not born in the Netherlands), and unknown.

In order to balance statistical power and group homogeneity, an optimal 1:1 nearest-neighbour matching algorithm without replacement was used, with a calliper of maximally 0.1 standard deviation on each of the covariates (Austin 2011b). In line with the guidelines reported by Baser (Baser 2006), five criteria were analysed to diagnose the balance properties of the matched sets. First, the continuous variables, age and baseline severity, were compared with a *t* test; and the categorical variables, gender, diagnosis group, and country of origin, were compared with a Chi square test. Second, standardized mean differences were calculated. Samples with a standardized mean difference of less than 0.1 on each covariate indicated negligible differences (Normand et al. 2001). Third, the percentage of reduction bias in the means was calculated for the variables, age and baseline severity. Fourth, a Kolmogorov–Smirnov test was used to compare the density estimates for the covariates, age and baseline severity (Conover 1999). Fifth, a Kolmogorov–Smirnov test was used to compare the density estimates of the produced propensity scores of the two matched samples.

Multiple linear regression was used to calculate the differences in cost and outcome between the two samples, with age, gender, baseline symptom severity, diagnosis group, and country of origin as covariates. The differences were expressed using the ICER.

The impact of the uncertainty of the regression coefficients on the ICER was assessed with a non-parametric bootstrap analysis of the package ‘boot’ (Davison and Hinkley 1997; Ripley and Canty 2016). This analysis samples a subset of the data 2500 times, and the differences in cost and outcome of different subsets of the population were estimated with a regression analysis. The bootstrapped outcomes of the ICER were presented in a cost-effectiveness plane and cost-effectiveness acceptability curve (van Hout et al. 1994).

### Sensitivity Analysis

Three assumptions were made to determine the cost per unit for each type of healthcare professional in both mental healthcare systems. These assumptions concern caregiver salary, number of billable hours, and overhead costs. The uncertainties in these assumptions were analysed by hypothetically varying the parameters to the disadvantage of basic mental healthcare. First, we increased the salaries of medical staff in basic mental healthcare by 10%. Then, we reduced the number of billable hours from 1300 to 1100. Finally, we increased the increment for overhead costs to 50% instead of 38%.

No assumptions were made in the estimation of treatment effect, and only empirical data was used to compare the outcome between the two mental healthcare systems.

## Results

### Sample Characteristics

A dataset of 11,867 patients with no missing information was available for the matching algorithm, which is about 50% of the total patient population of each organization that met the inclusion criteria.

Table 2 presents the characteristics of the two samples, of 4343 patients each, created by the matching algorithm. Four of the five criteria for a balanced set are met. First, the p values of each covariate indicate no significant differences between the means and distributions. Second, the standardized mean differences are below the threshold value of 0.1. Third, the calculated percentage in reduction bias in means of baseline severity (99.4) and age (93.1) are near 100 percent. Fourth, there is no significant difference between the density estimates of the produced propensity scores in the two matched samples [KS, D(4343) = 0.131, p = 0.849]. However, there is a significant difference between the density estimates of the covariates, age [KS, D(4343) = 0.044, p < 2.22e^−16^] and baseline severity [KS, D(4343) = 0.063, p < 2.22e^−16^].

**Table 2:** Sample size and observed patient characteristics after propensity score matching

	Specialized mental healthcare	Basic mental healthcare	SMD	p value
N	4343	4343		
Gender = F (%)	2762 (63.6)	2764 (63.6)	0.001	0.982
Age [mean (SD)]	38.0 (12)	38.1 (13.2)	0.009	0.682
Baseline severity [mean (SD)]	0.07 (0.98)	0.07 (0.92)	0.001	0.956
Diagnosis group = depression disorder (%)	2135 (49.2)	2183 (50.3)	0.022	0.313
Country of origin (%)			0.014	0.817
Netherlands	3444 (79.3)	3422 (78.8)		
Abroad	715 (16.5)	737 (17.0)		
Unknown	184 (4.2)	184 (4.2)		

Baseline severity is expressed as a Z-score; country of origin ‘Abroad’ was defined as not born in the Netherlands

*SMD* standardised mean difference

Table 3 presents the percentage of the treatment time provided by each type of healthcare professional in specialized and in basic mental healthcare, the resulting average treatment cost, and the average treatment effect, without adjustment for remaining confounding differences. The average unit cost for specialized mental healthcare is €75 and for basic mental healthcare is €83. The average number of treatment hours is 39.4 in specialized mental healthcare and 9.8 in basic mental healthcare.

**Table 3:** Discipline mix of healthcare professionals, mean treatment cost, and mean treatment effect (after propensity score matching)

	Specialized mental healthcare	Basic mental healthcare	SMD	p value
Other	8%	0%		
Clinical psychologist	2%	1%		
Social worker	5%	0%		
Basic doctor	6%	0%		
Psychotherapist	7%	2%		
Psychiatrist	8%	2%		
Nurse	8%	0%		
Psychiatric nurse	9%	0%		
Mental health psychologist	14%	70%		
Basic psychologist	34%	0%		
Unknown	0%	24%		
Other	8%	0%		
Cost [€ (SD)]	2952 (2788)	816 (362)	1.074	< 2.2e^−16^
Health outcome [d (SD)]	0.89 (1.02)	0.90 (1.02)	0.007	0.758

*SMD* standardised mean difference

### Cost-Effectiveness Analysis

Table 4 presents the outcome of the regression analysis and the incremental cost-effectiveness ratio. The table shows that treatment in basic mental healthcare occurs at significantly lower cost than treatment in specialized healthcare (− €2132; p < 2e^− 16^). Incremental health outcomes (expressed as effect size d) amount to 0.007. This difference in effect is not significant (p = 0.724) and not clinically relevant. With reduced costs and similar health outcomes, on average, the new healthcare system is dominant in terms of cost-effectiveness.

**Table 4:** Cost-effectiveness predicted with regression analysis

	Basic mental healthcare compared to specialized mental healthcare	Std Error	*t* value	p value
Additional cost	− €2132	42.328	− 50.373	< 2.0e^− 16^
Additional effect	0.007	0.021	0.352	0.725
ICER	Dominant			

*ICER* incremental cost effectiveness ratio

### Uncertainty Analysis

**Fig. 1:**
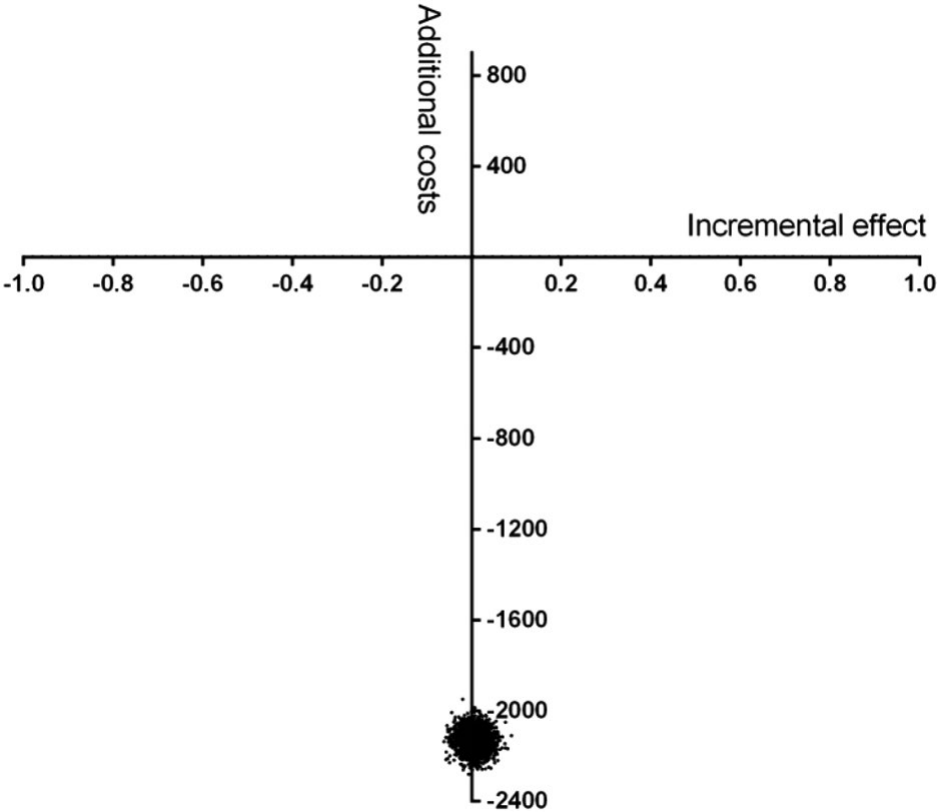
Cost-effectiveness plane for basic mental healthcare compared to specialized mental healthcare. Cost-effectiveness plane, presenting the 2500 bootstrap iterations in which the incremental cost-effectiveness ratio is estimated using a regression analysis. Effect in effect size D; costs are in euros

The impact on the ICER of uncertainty in our coefficients is shown in the cost-effectiveness plane (Fig. 1). The cost-effectiveness plane shows the outcome of the bootstrap analysis. The distribution of bootstrapped ICERs shows that 62% of the outcomes fall in the lower right-hand quadrant, which implies that the new system has a better health outcome at lower cost. For 38%, the ratio falls in the lower left-hand quadrant, which indicates that the new system has a worse health outcome at lower cost, though the maximum loss of effect is d = 0.06, which can be considered very small (Cohen 1988).

### Sensitivity Analysis

The average costs were calculated with a change in three different input parameters to the disadvantage of basic mental healthcare. Increasing the salary of basic mental healthcare professionals by 10%, reducing the number of billable hours in basic mental healthcare to 1100, or increasing the increment in overhead costs to 50%, does not change the conclusion of the analysis. The variation in billable hours has the most impact, and increases the average cost of basic mental healthcare to €963, which would reduce the difference in costs from €2133 to €1986.

## Discussion

In the short run, it seems that the treatment setting in basic mental healthcare contributed towards the aims of the healthcare reform, i.e. lower costs with equal or better outcome. This means that for a specific population, shortening treatment duration does not come at the expense of the effectiveness of the treatment. This is in line with the outcomes presented by Cuijpers et al. (Cuijpers et al. 2013), who used a meta regression analysis to show that there was no association between the number of therapy sessions and the effect size when using psychotherapy in the treatment of adults with depression. Finding similar effectiveness in these short treatments in basic mental healthcare may be due to the prior knowledge of both therapist and patient that treatment will be of limited duration. This knowledge may influence the behaviour of the patient as well as the therapist. Several studies show that the actual number of sessions is related to the number of sessions expected by patients (Mueller and Pekarik 2000; Owen et al. 2009). Moreover, the expectation of a short treatment compared to the expectation of a long treatment is related to a quicker response (Barkham et al. 1996). For a therapist, this limitation means that on the one hand the treatment has to be focused from the very start but on the other hand the therapist must actively encourage the self management and empowerment of the patient. Focusing, i.e. minimizing distractions and changes in treatment, increases the effectiveness of treatment (Schulte and Eifert 2002). This requires therapists who are specifically trained in such a focussed method, and who are able to empower the patient to take responsibility and self direction in his treatment.

It is interesting to note that, in our dataset, the average cost per hour in basic mental healthcare is actually higher (€83) than the average cost per hour in specialized mental healthcare (€75). Basic mental healthcare professionals are very well educated, but specialized mental healthcare professionals have a higher and more specialized education. However, basic mental healthcare is primarily monodisciplinary, i.e. healthcare professionals generally work alone, whereas specialized mental healthcare is multidisciplinary and includes a large proportion of staff with a lower level of education. The composition of the multidisciplinary teams in specialized mental healthcare corresponds with a lower average cost per hour than the monodisciplinary counterpart in basic mental healthcare.

Given the short duration considered in this analysis, the results are promising and potentially of high relevance to policymaking. Providing a duration-limited treatment to patients with anxiety or depression has the potential to decrease budget without losing health effects. The number of patients qualifying for such treatment is high, with an estimated 260,000 patients per year being treated (KPMG 2016) in basic mental healthcare in the Netherlands, and with an estimated 125,000 patients per year being treated for anxiety or depression, who were formerly being treated in specialized mental healthcare. Our preliminary results therefore suggest that this entails a savings potential of about 270 million euros per year in the Netherlands. As the transition was still in progress in the time period considered in this report, it is important to monitor the patient population treated in basic mental healthcare, such that future research can evaluate to what extent our sample is representative for patients treated in basic mental healthcare. All in all, the promising results in our analysis, and the magnitude of potential savings at a national level, justify giving priority to further research in this area. Furthermore, our approach could be used in more and broader settings, for instance to evaluate differences in healthcare between different regions or countries, thereby emphasizing best practice that could be used to further improve healthcare (de Beurs et al. 2015).

Future research is needed to further evaluate the system change. The analysis presented in this article provides a starting point for using administrative data to monitor the cost-effectiveness of such a system change. With more data available it would be possible to analyse potential relapse effects and to investigate whether the treatment effect of basic mental healthcare persists in the long run. On top of that, more data could be gathered about additional costs and benefits not directly related to mental healthcare treatment. With such data it is possible to analyse whether the new system is still cost-effective using a broader perspective. A randomized controlled trial similar to the study of Barkham et al. (Barkham et al. 1996) might not reveal the full impact of shortening treatment and changing the focus and responsibilities of treatment in a new setting. Therefore a cluster randomized controlled trial or a stepped wedge study should be used to confirm whether knowing at the time of starting treatment that its duration will be limited, results in lower costs with equal effects.

### Limitations

The main strength of our approach is the use of a large database of routine outcome monitoring data that allowed us to conduct a real-life experiment with historical matched cohorts from before and after a system change. Our study has a number of limitations, however, that need to be acknowledged.

First, as patients were not randomized, and only patients with no missing data were included, there is a risk of both overt and hidden selection bias. To minimize the risk of overt selection bias, patients in both groups were matched using the propensity score algorithm as outlined by Austin et al. (Austin 2011b). Additional checks on the quality of the resulting matched samples as recommended by Baser (Baser 2006) revealed that four of the five criteria were fulfilled. The criterion that was not fulfilled was the Kolmogorov–Smirnov test on the individual covariates, age and baseline severity, which is used to test whether these covariates in both samples, after matching, are drawn from the same distribution. Additionally, the Mann–Whitney test was used to test for similar distributions, showing that the two samples produced by the propensity score algorithm were likely to follow a similar distribution of the covariates, age [MW(4343), p = 0.327] and baseline severity [MW(4343), p = 0.432]. Moreover, one of the criteria that was fulfilled entailed a Kolmogorov–Smirnov test to indicate that both samples follow a similar distribution of the produced propensity score, which can be seen as a summary of the distribution of all covariates together (Austin 2011a). Furthermore, a regression analysis was used to adjust for remaining confounding differences. However, no technique [such as an instrumental variable approach (De Ridder and De Graeve 2011)] was used to deal with hidden selection bias.

Second, we compared the difference in cost and effect only over the duration of a completed treatment and not over a set period of time. Because the difference in cost is due to the difference in duration between the treatments in the two systems, it is possible that both cost and treatment effects are biased. We are unaware of how the treatment effects, occurring in the basic mental healthcare sample, persist after the end of these short treatments. Furthermore, we do not know how much care the patients in basic mental healthcare have consumed alongside and after their treatment. What we do know is that only about 5% of the patients in basic mental healthcare are referred to specialised mental healthcare after treatment termination (KPMG 2016).

Third, our analysis is limited to patients treated for anxiety and/or depression. It is unclear what the cost-effectiveness of the health system change is for patients treated for different disorders. Patients with anxiety and depression are estimated to make up the majority of patients treated in basic mental healthcare, but it is important to increase our understanding of the cost-effectiveness of the remaining patient population treated in basic mental healthcare.

Fourth, our analysis is limited to the Netherlands, and therefore the validity of our findings is limited to the specific context of the Dutch system in transition.

Fifth, a healthcare perspective was adopted, in which only data available from the reimbursement is used to compare intervention costs. For example, costs made for laboratory tests, medication, and hospitalization are not included in the analysis. Although we think that such resource usage is rare among the less complex patient population considered, a broader perspective, such as taking into account all healthcare costs, or taking into account societal costs such as productivity losses, could potentially alter our findings.

Sixth, even though the new situation seems preferable from a cost-effectiveness point of view, desirability should also be evaluated from different standpoints, such as equity, ethics, and sustainability (Berghmans et al. 2004; Mihalopoulos et al. 2011).

## Conclusion

The aim of this study was to compare the costs and effects for patients treated in basic mental healthcare with that for comparable patients treated in specialized mental healthcare. For the group of patients that was eligible for basic mental healthcare, the results suggest that treatment in the basic mental healthcare approach (after the reform of the mental healthcare system) was cost-saving. Treatment in basic mental healthcare showed a large significant reduction in costs, with similar health effects. Probabilistic uncertainty analysis showed that the average estimates of costs are robust, and that the potential loss in effect is negligible.

As the healthcare system in the Netherlands is in the midst of a reform, it is too early to draw conclusions on whether the new situation is to be preferred in terms of cost-effectiveness. Nevertheless, results at this stage are promising, showing the potential for generating similar effectiveness at lower costs for patients with anxiety and/or depression.
